# Acknowledgement to Reviewers of *Cancers* in 2014

**DOI:** 10.3390/cancers7010092

**Published:** 2015-01-07

**Authors:** Cancers Editorial Office

**Affiliations:** MDPI AG, Klybeckstrasse 64, CH-4057 Basel, Switzerland

The editors of *Cancers* would like to express their sincere gratitude to the following reviewers for assessing manuscripts in 2014:

Abdelmohsen, Kotb
Akimoto, Kazunori
Albiges, Laurence
Al-Ejeh, Fares
Alexeev, Vitali
Alison, Malcolm
Alvarez-Salas, Luis Marat
Aoki, Kazunori
Arriola, Edurne
Baba, Shingo
Bar-Natan, Michal
Barr, Martin
Beauchemin, Nicole
Becker, Jürgen
Beebe, Stephen
Bergmann, Lothar
Bernert, John
Bernier, Michel
Bielenberg, Diane
Bignami, Margherita
Binder, Mascha
Birchmeier, Walter
Birembaut, Philippe
Blandino, Giovanni
Bock, Jonathan
Bogliolo, Massimo
Boros, Laszlo
Bottaro, Donald
Breckpot, Karine
Brinkmann, Melanie
Brodt, Pnina
Bromberg, Jackie
Bros, Matthias
Brownell, Isaac
Bryan, Brad
Calorini, Lido
Calzavara-Pinton, Piergiacomo
Carmella, Steven
Cathomas, Gieri
Chandran, Bala
Chang, Yu-Jia
Chellappan, Srikumar
Chen, Yi-Ting
Cheng, Nikki
Chetty, Chandramu
Chiang, Chi-Shiun
Chiou, Shih-hwa
Cho, William
Choi, Kyung-Chul
Chouaib, Salem
Chuang, Shu-Chun
Chuma, Makoto
Collins, Sean
Conti, Alfredo
Costantini, Susan
Cristini, Vittorio
Culig, Zoran
Curtin, Nicola
Dai, Yun
Damia, Giovanna
Daniele, Antonella
Dean, Jonathan
Debatisse, Michelle
Decrock, Elke
Dedhar, Shoukat
Dienstmann, Rodrigo
Dieu-Nosjean, Marie-Caroline
Dings, Ruud
Dobner, Thomas
Dowlati, Afshin
Duda, Dan
Eisterer, Wolfgang
Erkizan, Verda
Erovic, Boban
Farah, Jad Walid
de Faria, Paulo Rogério
Ferlito, Alfio
Ferri, Clodoveo
Fields, Gregg
Fingleton, Barbara
Fischer, Nicole
Fitzgerald, Timothy
Franco, Rodrigo
Frank, Dale
Frank, David
Friedlander, Terence
Gabrielli, Brian
Gao, Ling
Gardner, Paul
Gavrilovic, Jelena
Gehrmann, Mathias
Georgakilas, Alexandros
Gerner, Christopher
Gessain, Antoine
Gewirth, Daniel
Gilles, Christine
Gires, Olivier
Giuliani, Nicola
Goldberg, Gregory
Golemis, Erica
Gorgoulis, Vassilis
Grade, Marian
Gröbe, Alexander
Groner, Bernd
Guin, Sunny
Gullberg, Donald
Hadrup, Sine Reker
Hagedorn, Martin
Halazonetis, Thanos
Hanin, Leonid
Haque, Azizul
Hardingham, Jennifer
Hata, Yutaka
Hatt, Mathieu
Haystead, Timothy
Hayward, S. Diane
Heckman-Stoddard, Brandy
Heery, David
Held, Kathy
Helmbold, Peter
Hiraku, Yusuke
Ho, Heng-Chien
Hockenbery, David
Hodny, Zdenek
Hoppe, Bradford
Hori, Tomohide
Houghton, A. McGarry
Howle, Julie
Hruby, George
Hu, Cheng-Jun
Hu, Yinling
Hurst, Douglas
Izumiya, Yoshi
Jager, Martine
Jala, Venkatakrishna Rao
Jonassen, Chrinstine
Jonckheere, Nicolas
Kalady, Matthew
Kaminska, Bozena
Kanda, Tatsuo
Kasperkiewicz, Michael
Kato, Ikuko
Kenneth, Kin Wah
Kevin, Gaston
Kim, Won
Kimple, Randall
Klingelhutz, Aloysius
Knecht, Hans
Koljonen, Virve
Komohara, Yoshihiro
Kortylewski, Marcin
Krasagakis, Konstantin
Kubiseski, Terrance
Kueh, Hao Yuan
Kuo, Min-Liang
Kuperwasser, Charlotte
Lace, Michael
Lam, Yee Cheong
Landolfo, Santo
Lang Kuhs, Krystle
Lantz, Olivier
Larner, Andrew
Lathia, Justin
Le Rhun, Emilie
Lee, Dae Ho
Lee, Nam
Li, Min
Li, Qing Kay
Li, Zuofeng
Lianidou, Evi
Lin, Ho
Liška, Václav
Liszka, Łukasz
Liu, Gang
Liu, Qi
Lo Muzio, Lorenzo
Lo, Kwok-Wai
Looyenga, Brendan
Lopez-Martinez, Giancarlo
Lukas, Rimas
Ma, Lijun
Ma, Shuang
Machesky, Laura
Machida, Keigo
Madsen, Steen
Magee, Pamela
Magnusson, Lisa
Mak, Nai
Malemud, Charles
Marabese, Mirko
Massarweh, Suleiman
Massimo, Tommassino
Mehta, Rajendra
Melendy, Tom
Melisi, Davide
Michaelson, James
Michiels, Carine
Minegishi, Yuji
Mori, Naoki
Morrione, Andrea
Muller, Patricia
Naehrig, Diana
Nakajima, Koichi
Nasser, Mohd
Natrajan, Rachel
Newton, Alexandra
Nickoloff, Jac
Nicola, Nicos
Nixon, Andrew
Ogris, Manfred
Okazaki, Isao
Olivier, Magali
Opdenakker, Ghislain
Ospina, Juan David
Ouadid-Ahidouch, Halima
Ozawa, Masayuki
Pappas, Dimitri
Pavia, Maria
Peker, Deniz
Peña, Maria Marjorette
Penichet, Manuel
Pertsemlidis, Alexander
Petak, Istvan
Peterson, Lanell
Pichiorri, Flavia
Pilati, Camilla
Pinsky, Paul
Pitzalis, Costantino
Polette, Myriam
Poli, Valeria
Ponnambalam, Sreenivasan
Poon, Randy
Porteu, Francoise
Prabhu, Soorebettu
Prato, Mauro
Quan, Taihao
Raab-Traub, Nancy
Reddy, Sakamuri
Renò, Filippo
Rich, Jeremy
Riesterer, Oliver
Rivas, Carmen
Roberts, David
Robertson, Erle
Rodríguez, Jose
Roeb, Elke
Rolny, Charlotte
Rosell, Rafael
Ruggeri, Rosaria
Rutenberg, Michael
Sakamoto, Kaori
Sampson, John
Santarpia, Carmen
Sargi, Zoukaa
Sasano, Hironobu
Schemmer, Peter
Scher, Howard
Schmitt-Graeff, Annette
Schuessler, Andrea
Schwertfeger, Kaylee
Semenza, Gregg
Serini, Guido
Shackelford, David
Shen, Chiung-Chyi
Shi, Jiaqi
Shih, Wen-Ling
Shintani, Yasushi
Shurin, Michael
Siebenhaar, Frank
Siemann, Dietmar
Simcox, Mary
Simiele, Felice
Singh, Ishwar
Singh, Seema
Smith, Steven
Sonveaux, Pierre
Soroceanu, Liliana
Sourvinos, George
Sprung, Carl
Stagni, Venturina
Steffens, Sandra Steffens
Steinau, Martin
Strongin, Alex
Tabocchini, Maria Antonella
Takahashi, Masahide
Taly, Valerie
Tanaka, Takuji
Tapon, Nicolas
Tarasova, Nadya
Torigoe, Toshihiko
Torrejón, Davis
Toth, Zsolt
Touze, Antoine
Traina, Tiffany
Treglia, Giorgio
Trovato, Maria
Tseng, Ching-Ping
Turkson, James
Tybulewicz, Victor
Ueno, Naoto
Uhr, Jonathan
Vaira, Valentina
van Hall, Thorblad
Vandooren, Jennifer
Verhaegen, Monique
Veronesi, Giulia
Viola, Giampietro
Vogel, Lotte
Vona-Davis, Linda
Walsh, Noreen
Wang, Guiren
Wang, Huan-You
Wang, Qing
Wang, Yu-Chieh
Ware, Carol
Warren, Graham
Watts, Colin
Weitzman, Matthew
Wen, Yangan
Wendtner, Clemens-Martin
Whatcott, Clifford
Whitehouse, Adrian
Wieczorek, Edyta
Wilson, Van
Wyss, Annah
Xia, Jing
Xia, Yang
Xie, Guofeng
Xu, Keli
Yenari, Midori
Yoshizaki, Tomokazu
You, Liang
Young, Jason
Yu, Jun
Zhang, Mingliang
Zhang, Yan
Zhang, Yu-Wen
Ziman, Mel

